# Infection causes childhood leukemia

**DOI:** 10.18632/aging.100815

**Published:** 2015-09-26

**Authors:** Julia Hauer, Alberto Martín-Lorenzo, Isidro Sánchez-García

**Affiliations:** Instituto de Biologia Molecular y Celular del Cancer (IBMCC), CSIC/Universidad de Salamanca, Campus M. de Unamuno s/n, 37007-Salamanca

**Keywords:** leukemia, infection, murine models, genetic susceptibility

Leukemia accounts for a third of all cancers in children under the age of 15, mostly affecting industrialized countries, being the B-precursor acute lymphoblastic leukemia (pB-ALL) the most common type. The disease commonly strikes young children at the age of two to five years, and is treated with aggressive chemotherapy. In recent decades, more effective treatments have boosted survival rates and reduced mortality for childhood leukemia. However, according to SEER data from the National Cancer Institute, the incidence of lymphoblastic leukemia (ALL) in 0-14 year-old US children increased from 2.2 per 100,000 in 1975 to 4.0 per 100,000 in 2005. This might be caused in part due to better cancer registries but there is little doubt that the real incidence of this “modern life-style” malignancy also rose. Hence, preventive strategies are clearly superior to any therapy improvement. Environmental exposures came into the focus of many epidemiologists who initiated a number of well-conducted studies aiming to elucidate the etiological role of infection, nuclear power-plants, background terrestrial irradiation and many other putative factors.

Over the past century, infections have been regarded as the most likely cause of childhood leukemias [1]. But in general, epidemiological studies are descriptive and sometimes vary in their main conclusions. Similarly, when wild type mice were born and kept in the specific-pathogen-free (SPF) environment until moved to common infectious environment they do not to develop pB-ALL [2]. These results suggest that delayed-exposure to infection is not involved in the leukemia etiology. However, it is potentially possible that, in humans, infection-exposure favors pB-ALL in children harboring an intrinsic genetic susceptibility. Recently, germline mutations in *PAX5* have been described as conferring an inherited risk for pB-ALL and pB-ALL occurs in affected children at reduced penetrance [3, 4].

**Figure 1:**
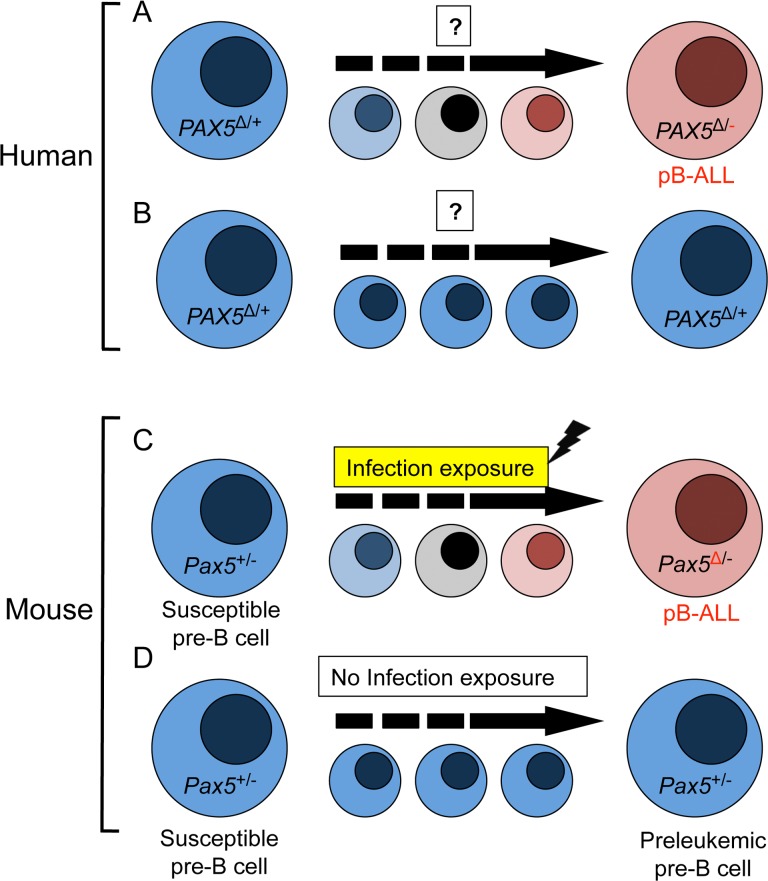
Exposure to infection is a causal factor in B-precursor acute lymphoblastic leukemia as a result of Pax5 inherited susceptibility (**A**) In humans, *PAX5* c.547G>A predisposes to pB-ALL. Retention of *PAX5* hypomorphic allele and deletion of the wild type *PAX5* allele is observed in about 30% of *PAX5* c.547G>A carriers and results in pB-ALL development. All leukemia show deletions of chromosome 9p. However, knowledge about preleukemic cell populations and environmental exposure is not yet available in carrier families. **(B)** Without somatic secondary genetic events – e.g. loss of the wt *PAX5* allele - no pB-ALL develops (**C**) *Pax5*+/− mice acquire point mutations in the second allele of *Pax5* when they are exposed to infection and this triggers pB-ALL development. (**D**) *Pax5*+/− mice do not develop leukemia under non-infection exposure conditions. **ß**: point mutation; **+**: wild type *Pax5* allele; **-**: deletion of *Pax5* allele.

To elucidate if pB-ALL is a result of infection exposure, *Pax5+/−* mice were exposed to common pathogens after being born in SPF condition. *Pax5+/−* mice developed clonal pB-ALL recapitulating the clinical, histopathological and molecular features of human pB-ALL. These data represent the first proof that delayed exposure to infection can induce human-like pB-ALL in mice on the basis of inherited genetic predisposition, suggesting that a similar scenario may occur in human pB-ALL [2]. The demonstration that pB-ALL development can be established in mice by delayed exposure to infection implies that either abolishing infection or exposure to infection very early after birth will interfere with pB-ALL generation (Figure 1). In agreement with this, *Pax5+/−* mice kept under SPF environment during their lifespan did not spontaneously develop pB-ALL [2].

But the most crucial question is, how does infection exposure give rise to pB-ALL? In order to identify the mechanism of this specific pB-ALL susceptibility due to *Pax5* heterozygosity under exposure to infections, we characterized both B-cell development in healthy and B-cells in leukemic *Pax5+/−* mice [2]. The data showed that monoallelic loss of *Pax5* promotes leukemogenesis by creating an aberrant IL7-sensitive progenitor compartment. This pre-leukemic preB cell population is susceptible to malignant transformation through accumulation of secondary *Jak3* mutations, which depicts a rescue mechanism of the IL7/IL7R/STAT5 signaling. Transplantation experiments demonstrate that the activating *Jak3* mutations per se are sufficient to drive leukemia. Thus targeting the deregulated JAK/STAT pathway can be a promising therapy for this disease.

Patients with a genetic predisposition (Pax5 c.547G>A) loose the WT *PAX5* allele related to a secondary structural aberration of chromosome 9p. In mice we face the same scenario but the sequence is reverse. The mice lack one *Pax5* allele and acquire *Pax5* point mutations on the remaining WT allele as a secondary event. The net result in both species is reduced transcriptional activity of Pax5 (Figure 1). The mechanisms responsible for the conversion of the preleukemic clone, carrying the inherited mutations of *PAX5,* into pB-ALL are not understood yet. However, recent results suggest that the B cell-specific enzyme AID is supposed to be the predominant driver of clonal evolution in human ETV6-RUNX1 pB-ALL [5, 6] and B-cell lymphoma [7]. The pB-ALL in our mouse model originated as a result of delayed infection exposure. It offers a unique possibility to confirm if the proposed AID-mechanisms are involved in the conversion of the preleukemic clone into a full-blown leukemia.

Other major question that arise in light of these findings are if the timing and pattern of infectious exposure is indeed relevant for pB-ALL development, how the second hit impacts on the target cell, and what are the qualitative and/or quantitative figures that make *Pax*5+/− stem/progenitor target cells more vulnerable to malignancy. At the end, it will broaden our horizon and help us to change the way we approach childhood leukemia, from diagnosis and treatment to prevention first.
